# Quantitative Analysis of Bioluminescence Optical Signal

**DOI:** 10.3390/bios13020223

**Published:** 2023-02-03

**Authors:** Kazuki Niwa, Hidehiro Kubota, Toshiteru Enomoto, Yoshiro Ichino, Yoshihiro Ohmiya

**Affiliations:** 1 National Institute of Advanced Industrial Science & Technology (AIST), Tsukuba 305-8563, Japan; 2 ATTO Corporation, Tokyo 110-0016, Japan; 3 Osaka Institute of Technology (OIT), Osaka 535-8585, Japan

**Keywords:** bioluminescence, imaging, optical signal, optical reference light source, standardization

## Abstract

Bioluminescence is light emission based on the luciferin–luciferase enzymatic reaction in living organisms. Optical signals from bioluminescence (BL) reactions are available for bioanalysis and bioreporters for gene expression, in vitro, in vivo, and ex vivo bioimaging, immunoassay, and other applications. Although there are numerous bioanalysis methods based on BL signal measurements, the BL signal is measured as a relative value, and not as an absolute value. Recently, some approaches have been established to completely quantify the BL signal, resulting in, for instance, the redetermination of the quantum yield of the BL reaction and counting the photon number of the BL signal at the single-cell level. Reliable and reproducible understanding of biological events in the bioanalysis and bioreporter fields can be achieved by means of standardized absolute optical signal measurements, which is described in an International Organization for Standardization (ISO) document.

## 1. Introduction: Why Do We Have to Quantify the Optical Signal?

Optical signals from bioluminescence (BL), chemiluminescence (CL), and fluorescence have been used in analytical methods to measure biological samples in the fields of life science and medicine. Components of biological samples or biological parameters, including cellular and metabolic activities, and gene expression, are transformed into an “optical signal”, and the mechanism or function at the cell, tissue, or body levels is investigated. One of the optical signals is BL, which is based on the chemical oxidation of luciferin catalyzed by the luciferase enzyme. The intensity of the optical signals on the BL is determined by the efficiency of the enzymatic reaction and the number of luciferins and cofactors. For instance, the optical signal of firefly BL is determined by the amount of luciferin, luciferase, and adenosine triphosphate (ATP), as well as the enzyme efficiency, including the quantum yield (QY) of the luciferin–luciferase reaction [1,2]. 

Ten luciferins have been identified in bioluminescent organisms, along with several corresponding luciferases [3]. Firefly D-luciferin, coelenterazine, *Cypridina* luciferin, and their derivatives have been commercialized, and are primarily used in reporter assays, in vivo and in vitro imaging, and immunoassay [4,5,6,7,8,9]. Several companies, such as Promega and Toyobo, have also commercialized luciferase genes.

The firefly luciferin–luciferase reaction has mainly been applied to in vitro gene expression analysis as a reporter gene assay for screening bioactive compounds or toxicants [10,11,12,13,14]. This reaction has also been used to evaluate in vitro cellular functions as well as in vivo cancer growth and tracing [15,16,17,18]. Coelenterazine-based luciferase reactions have been used for gene expression analysis and in vivo imaging [19,20,21,22,23]. *Cypridina* luciferin–luciferase reactions have also been used in gene expression analysis, in vivo imaging, and immunoassays [24,25,26,27,28,29,30]. 

We constructed a reporter plasmid vector containing the target promoter and luciferase gene sequences for the gene expression analysis reporter assay. The promoter region regulates the expression of the luciferase gene in living cells after transfection of the plasmid into the target cells. The expressed luciferase protein catalyzes a reaction with luciferin to produce an optical signal, resulting in the evaluation of changes in promoter activities influenced by factors such as compounds. Therefore, it is possible to identify active compounds from natural resources and detect toxicants from chemicals. 

The promoter region was found to regulate the expression of luciferase genes in living cells for in vitro imaging after transfection of a plasmid containing the luciferase gene into the target cells. For the imaging experiment, luciferin was added to the medium, penetrated the cells, and reacted with the expressed luciferases to generate light emission, resulting in the visualization of the events of living cells. For in vivo imaging, after transfection of the plasmid containing the luciferase gene into target tumor cells, we selected stable cell lines expressing luciferase, followed by transplantation into model animals. For in vivo imaging experiments, luciferin was injected into the body after an appropriate period for tumor cell growth to generate an optical signal. BL signals indicate the location and growth of tumor cells in the body. BL imaging (BLI) is measured using special equipment and is indicated by brightness in the image. 

Luciferase-fused antibodies are used in sandwich or competitive immunoassays, the so-called bioluminescent enzyme immunoassay (BLEIA) [31,32]. By constructing a calibration curve, the target antigen bound to a luciferase-fused antibody can be quantified with high sensitivity and a large dynamic range. 

The optical signals of BL methods are measured by sensitive photodetectors, including photomultipliers, photodiodes, CCD, and CMOS sensors, and are converted into the amount of target molecules based on the calibration curve between the light intensity and the number of target molecules. In particular, immunoassays using BL are highly quantitative and reliable and have been commercialized by many companies. However, the measured optical signals are relative values; therefore, the detected optical signals collected on different days using different equipment or luminescent probes cannot be compared directly.

BL signals by reporter-gene assays or BL imaging have mainly been shown as intensity or brightness in relative light units (RLU). However, the relative intensities of the BL signal cannot be compared quantitatively between blue and red light, because the detection efficiency of the photodetectors depends on the wavelength of the incident light. To effectively use BL methods, the BL signal must be measured using a new quantitative measurement procedure.

## 2. What Is an Absolute Optical Signal? How Is an Absolute Optical Signal Quantified?

In bioluminescence-based biological analysis methods, optical signals are emitted from biological samples owing to the BL reactions. The absolute values of the BL signals can be interpreted as the power of the light flux or the number of quantum photons emitted from the biological samples. In radiometry, the power of the optical signal is described as the total radiant flux (W), and the quantum photon flux is the total photon flux (photons s^−1^). The energy of a single quantum photon can be described as follows:

*E* = *hν* = *hc*/*λ*, (1)

where *E* is the energy of the quantum photon, *h* is Planck’s constant, *ν* is the frequency, *c* is the velocity of the light, and *λ* is the wavelength. Thus, spectrometric information is also mandated with total radiant flux (W) information to provide the total photon flux (photons s^−1^).

The responsivity of the photodetectors can be calibrated to measure the absolute optical signal values of the samples. Here, we define the “responsivity” of the photodetectors as the sensitivity (e.g., in counts photon^−1^) to the incident radiant flux or photon flux. The responsivity of photodetectors is usually calibrated by manufacturers using the linear flux of directional monochromatic light sources at each representative wavelength. However, BL optical signals from biological samples are not directional, but diffusive and non-directional, unlike lasers. Therefore, the optical signal emitted from a biological sample is partially detected by the photodetector in the device, which includes a luminometer, plate reader, and microscope. It is also true that the wavelength spectrum of a BL optical signal is broad and often consists of multiple spectral components, whereas lasers emit single- and narrow-wavelength light. 

Thus, the absolute responsivity of a device depends on the spatial angular distribution profiles of the optical signal emission as well as the wavelength spectrum of the optical signal from the biological sample under examination. Therefore, information on the absolute responsivity of the photodetector in a device is insufficient to determine the responsivity of the entire device. The absolute responsivity of the device for measuring the BL optical signal can be determined using a calibrated reference light source. The reference light source to be employed should have angular distribution profiles similar to those of the biological sample under examination as well as a power level within the detection range of the target device. Spectral matching is also essential when the device is not spectrometric because its responsivity depends strongly on the spectrum to be measured. The best reference light source for the absolute calibration of the BL optical signal measurement device was the same BL sample to be tested with a known calibrated value, referred to as the “optical reference light source”.

**Figure 1:**
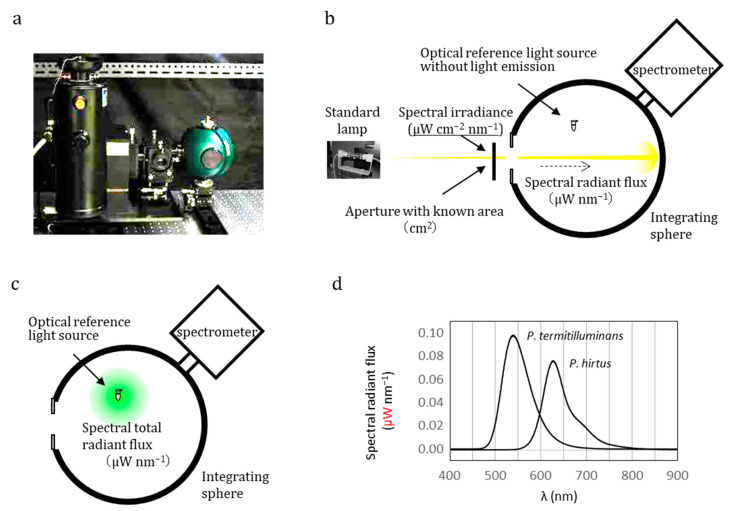
**Integrating sphere equipped with a spectrometer to measure the absolute optical signal value of optical reference light source.** (**a**) Integrating sphere spectrometer with a standard lamp. (**b**) Schematic illustration of calibration of the absolute sensitivity of the apparatus based on a standard lamp whose spectral irradiance value (μW cm^−2^ nm^−1^) and aperture area (cm^2^) are known. (**c**) Measurement of spectral radiant flux (μW nm^−1^) of luminescence solution. (**d**) Example of the absolute spectrum of an optical reference light source (bioluminescence reaction solution using native luciferases from *Pyrearinus termitilluminans* and *Phrixothrix hirtus*). Note that the y-axis describes an absolute value: spectral total radiant flux (μW nm^−1^). Using the equation *E* = *hν* = *hc*/*λ*, spectral total radiant flux can be transferred into spectral total photon flux (photons nm^−1^ s^−1^). Wavelength integration of the spectral total photon flux gives the total photon flux (photons s^−1^), which can be applied to calibrate of the absolute responsivity of luminometers.

The absolute value, that is, the total radiant flux (W) or total photon flux (photons s^−1^), of the optical reference light source can be measured using an integrating sphere spectrometer [33], as shown in Figure 1a. An integrating sphere equipped with a spectrometer can measure the absolute value of light sources with both directional and non-directional geometries. The absolute responsivity of the integrating sphere spectrometer was calibrated using a calibrated standard lamp of spectral irradiance (μW cm^−2^ nm^−1^), as shown in Figure 1b. The standard lamp used was traceable to the national standards of photometry and radiometry at the National Metrology Institute of Japan in National Institute of Advanced Industrial Science & Technology (NMIJ/AIST). The target optical reference light source was placed in a sphere to measure the spectral total radiant flux (μW nm^−1^) (Figure 1c). 

An ideal optical reference light source has long-lasting and stable optical signal intensity properties. However, the optical signal intensity from the optical reference light source tends to be influenced by the quality of the reagents, and hence has poor reproducibility. The reference solution was duplicated by dividing the homogeneous reaction solution into two test tubes [33]. One of them was set in the integrating sphere system, and the other was set in the device to be calibrated. By simultaneously measuring the homogeneous optical reference solution sample in the integrating sphere and the device to be calibrated, the absolute total radiant flux (μW) determined by the integrating sphere and the output value (e.g., in counts) measured by the device to be calibrated provide the absolute responsivity (counts μW^−1^) of the device. Using Equation (1), spectral total radiant flux of the optical reference light source can be transferred into the spectral total photon flux (photons nm^−1^ s^−1^), followed by the wavelength integration to give total photon flux (photons s-^1^) (Figure 1d). Relations between the total photon flux value and the output value (count) of a luminometer is the photon number-based absolute responsivity (counts photon^−1^).

Thus, a calibrated device is available for absolute measurement as long as the optical signal of the sample has spectral and spatial properties identical to those of the optical reference light source. The quantum yield (QY) of the BL reaction can be investigated as an application of absolute BL measurements using absolutely calibrated luminometers.

## 3. Determination of Quantum Yield on Luminescence Reaction

The QY of the BL reaction, which is defined as the probability of single-photon production from a single reactant molecule, is a basic characteristic for interpreting the molecular mechanism of luminescence reactions. To determine the QY of the BL reaction, the total number of photons emitted diffusively from the reaction solution, or total photon flux (photons s^−1^), must be measured. For this purpose, the optical signal measurement device must be calibrated.

In 1959, Seliger and McElroy reported a value of 0.88 using a North American *Photinus pyralis* native luciferase [34]. In 1962, Shimomura and Johnson reported the QY of *Cypridina* luciferin–luciferase reaction to be 0.28 [35]. In 1965, Lee and Seliger reported a QY value of 0.012 for a luminol chemiluminescence reaction [36]. After this report, the QY value of luminol was re-examined, and it converged to approximately 0.01, which was used as a reference to validate the optical apparatus for measuring the absolute photon flux from the luminescence reaction solution [37]. In 1986, Shimomura et al. also reported the QY of a photoprotein Aequorin to be 0.23 [38,39], but this value was corrected to 0.17 by Shimomura as the molecular weight of the photoprotein was revealed [40]. The QY of the BL reaction of coelenterazine and *Renilla* luciferase was reported by Loening et al. to be 0.069 [41]. 

The QY value of 0.88 in the firefly BL reaction reported in 1959 has long been cited as evidence of the most efficient luminous reaction. However, this report was published before the chemical structure determination of firefly luciferin molecules, and the synthesized compound was unavailable [42]. Therefore, in 1959, only native luciferin samples purified and isolated from firefly bodies were available. Notably, firefly luciferin is a chiral compound, and White et al. reported that a purified native luciferin sample was racemized [43]. This indicates that the QY value of 0.88, reported in 1959, would be corrected to an impossible value of 1.76, because native racemized luciferin was provided for the QY measurement, and only one of the optical isomers was active as the bioluminescence substrate. Nevertheless, the QY value of 0.88 was the only reported value for a long time that was based on the original experimental data. Therefore, it is important to re-examine the QY value of *P. pyralis* luciferase. Furthermore, there are various luminous beetles with different BL colors despite using identical firefly luciferin substrates [44]. Studies using luciferases from curious luminous beetles are in strong demand.

Ando et al. reported a re-examination of the firefly bioluminescence reaction quantum yield in 2008, which was 0.41 [45], differing significantly from the value of 0.88 reported in 1959. Ando et al. used a multichannel spectrometer whose spectral responsivity was calibrated in conjunction with a geometric treatment to precisely determine light collection efficiency [46]. QY values of firefly luciferin analogues were also reported using the same instrumentation [47].

To crosscheck the calibration system, the luminometers should be calibrated using an independent calibration system based on different measurement traceability sources. An integrating sphere spectrometer system was used to calibrate a commercially available luminometer against the absolute optical signal value of the firefly BL reaction solution [33]. The QY value of the same *P. pyralis* BL reaction solution was measured using a luminometer calibrated absolutely using the integrating sphere, and the result was 0.48, which was in agreement with the value reported by Ando et al. within the uncertainty of the measurements. 

We also measured the quantum yields of various beetle BL reactions using the same method (Table 1) [33,48]. Quantitative analysis of firefly bioluminescence reactions using various enzymes revealed that the kinetic parameters, such as *K*_m_, *k*_cat_, and QY, of native luciferases were comparable. This result indicates that the concentration of the “active” luciferase enzyme was the most important factor in obtaining high optical signal intensity. This result suggests that a better result with high optical signal intensity in BL reaction applications would be obtained by crucial efforts to optimize the reaction conditions for luciferase enzymes, each of which has different ideal conditions for the BL reaction. It has also been suggested that more rigid enzymes are more useful for applications because stable enzymes can maintain enzymatic activity.

For comprehensive quantitative understanding of BL reaction efficiency, further investigations have been conducted, e.g., QY of firefly BL reaction in biomolecular condensates [49]. We also investigated the QY using coelenterazine and its derivatives [50]. Notably, the highest QY value, 0.61, was observed when firefly luciferin was used with luciferase from the Brazilian click beetle *Pyrearinus termitillumineans*. Although the extremely high QY value of 0.88 was corrected, the QY value of 0.61 is still the highest among all luminescence reactions. To date, the firefly BL reaction remains the most efficient light-production system.

## 4. Establishing an Ultra-Weak Light Source as an Optical Reference

For QY investigation using absolutely calibrated luminometers, the BL reaction solution was employed as an optical reference light source with spectral and geometrical properties identical to those of the target BL sample. However, the BL reaction solutions were not stable or not reproducible. Therefore, they were not suitable optical reference light sources for general users of biological analysis methods using BL and CL. Ultra-weak light sources based on light-emitting diodes (LED) are already commercially available as optical reference light sources for checking the reproducibility and intermediate precision of BL and CL measurement devices for quality control purposes. Optical references are also recommended in an international standard document (ISO 24421) to verify the reliability of biological analysis methods using BL, CL, fluorescence, and absorption [51,52].

Optical reference light sources must only be reproducible in terms of the optical signal intensity to check the reproducibility of the devices. Therefore, absolute calibration is not mandatory. However, all devices must always be checked using optical references with the same optical properties because different references may have different optical signal intensities. Therefore, absolute optical signal measurements are required to confirm the uniformity of LED-based optical reference light sources. 

**Figure 2:**
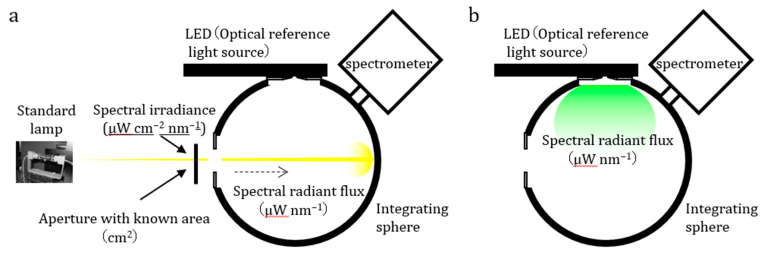
**Integrating sphere equipped with a spectrometer to measure the absolute optical signal value of LED-based optical reference light sources.** (**a**) Calibration of the absolute sensitivity of the apparatus based on a standard lamp whose spectral irradiance value (μW cm^−2^ nm^−1^) and aperture area (cm^2^) are known. (**b**) Measurement of spectral radiant flux (μW nm^−1^) of LED-based optical reference light source where wavelength integration of spectral radiant flux (μW nm^−1^) gives radiant flux (μW).

An integrating sphere can also be used to measure the absolute value of total radiant flux (μW cm^−2^ nm^−1^) of LED-based optical reference light sources that have a surface emission geometry (Figure 2). Absolutely calibrated LED-based optical reference light sources are available not only for quality control purposes, but also for quantitative imaging applications. 

To design a suitable optical reference light source to calibrate the imaging apparatus, several specifications must be considered: (1) a low-level and wide-range power (µW to fW) of light; (2) a planar emission surface to be set on the image plane or the sample position of BL measurement systems; (3) an emission area with sizes comparable to those of various samples (mm to µm); (4) a simple angular emission pattern, such as Lambertian or hemispherical; and (5) a long lifetime. Considering the above points, regarding the emission color or spectrum of BL samples, Yoshita et al. designed and developed planar LED devices, which were applied as optical reference light sources for quantitative measurements and analyses of photoluminescence (PL) intensity [53]. They demonstrated and confirmed the utility and applicability of an LED-based optical reference light source for quantitative luminescence intensity measurements in Lambertian-type low-level radiation sources.

**Figure 3:**
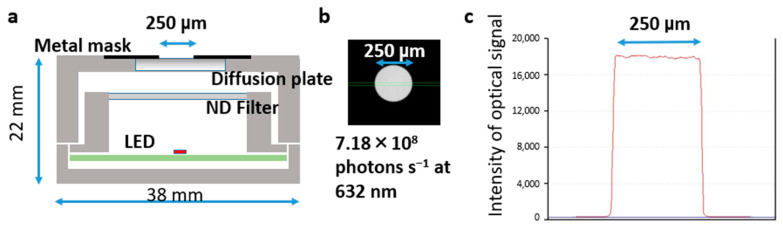
**Construct of the LED-based optical reference light source (ref. [53]).** (**a**) Schematic illustration of the planar light emitting diode (LED) device with a circular aperture in cross-sectional views. (**b**) Absolute optical image of the planar LED with an aperture size of 250 μm. This LED showed an absolute optical signal of 7.18 × 10^8^ photons s^−1^ at 632 nm. (**c**) Dispersion profile of absolute optical signal of the planar LED with an aperture size of 250 μm.

Based on the concept of an LED-based optical reference light source using low-level solid radiant sources, ATTO Corporation developed the planar optical reference light source “KohshiUni [54]”. Figure 3a shows a schematic of KohshiUni, which consists of a small surface-mounted LED chip, ND filter, and light diffusion plate coated with metal. The photoetching technique was used to create a 250 μm circular pinhole in the coated metal. Light was emitted through the pinhole. Figure 3b shows an image of the LED-based optical reference light source captured using the CCD camera unit. Figure 3c shows the profile of the optical signal on the surface of the planar optical reference light source, suggesting a simple uniform emission pattern. For device specifications, the light output power can be adjusted according to the target by changing the output power of the LED (pW level), ND filter, and emission area size. Furthermore, this device can be placed on the image plane or sample position of the BL measurement system. Finally, the absolute photon flux of this device was 7.18 × 10^8^ photons s^1^ at 632 nm, as evaluated by the integrated sphere spectrometer. Based on the relations between the relative conversion efficiency and wavelength in the CCD camera, detected photon number can be introduced from the values at 632 nm.

The BL optical signal depends on various systems based on the different colors and QY of the chemical reaction. However, the number of photons produced by the BL reaction can be converted into the number of luciferins. An LED-based optical reference light source can convert the relative light unit to the absolute photon number. When we use an LED-based optical reference light source in BL measurement, we can directly compare the optical signals in various BL systems.

## 5. Quantification of Bioluminescence Optical Signal from the Living Cells

In 1981, Trube et al. monitored the increase in calcium ions in living muscle tissue using the calcium-binding photoprotein aequorin [19]. In 2000, Rehemtulla et al. established a stably expressed firefly luciferase in a 9L cancer cell line and transplanted it into living mice, demonstrating the possibility of tracing cancer cell growth and evaluating anticancer drugs in vivo [15]. To date, chronological research using BL optical signals in living cells has been the most exciting research area. Wilsbacher et al. established the clock gene *Per*1-promotor driving optical signals in trans-genetic living mice and visualized the circadian rhythmicity of the *Per*1 gene in living tissue over one week [55]. In addition, this group established a *Per*1-promoter driving optical signal in Rat-1 cells and visualized circadian rhythmicity at the single-cell level [56]. Noguchi et al. monitored two clock genes of *Per*1 and anti-phased *Bmal*1 genes by using green- and red-emitting beetle luciferases at the cell population level [57]. 

In contrast, the improvement of several types of luciferases, such as color difference beetle luciferases, secreted-type luciferases, and enhanced *Renilla* luciferase, has been used to visualize various cell functions in living cells. The green- and red-emitting beetle luciferases visualized the circadian rhythmicity of two clock genes, *Per*1 and *Bmal*1, in a single cell [58] and the dynamics of the expression of the two genes at the subcellular level [59]. Secreted *Cypridina* luciferase was used to visualize the secretion process of proteins [25] and evaluate the potential of the signal sequence in the secretion process [60]. Enhanced GFP-fused *Renilla* luciferase visualizes chromatin movement in living single cells [22]. Enhanced Nano-lantern luciferase visualizes cell function in living single cells [61]. Gregor et al. established an autonomous bioluminescence mammalian cell using a bacterial bioluminescence system and visualized cell morphologies at the single-cell level [62]. In particular, the bioluminescence imaging of the chronological system contributed to the Nobel Prize in 2017.

We used highly sensitive cooled CCD, EM-CCD, and CMOS cameras to visualize the light intensity of the optical signal. First, the imaging equipment must be calibrated with an optical reference light source to quantify the optical signal from several detectors with varying color sensitivities. Enomoto et al. used an LED-based optical reference light source with pulse-width modulation (PWM) and a light-emitting aperture at an emission wavelength of 632 nm, which stabilized the light output over time [63]. It is important to evaluate the total radiant flux (W) of this light source, which is linked to a national metrology institute (for instance, NMIJ/AIST of Japan), based on the absolute sphere method. The procedure is summarized as follows.

**Figure 4:**
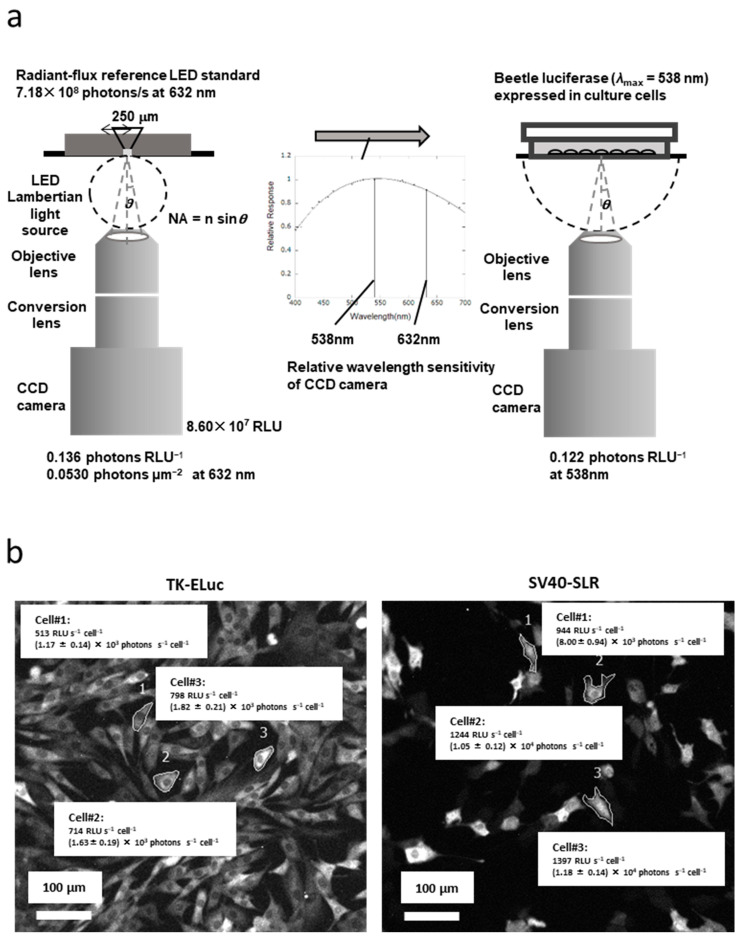
**Outline of absolute bioluminescence imaging from procedure to results (ref. [63]).** (**a**) Schematic illustrations of the setup and procedure for the calibration of the spectral response of the bioluminescence imaging system based on the LED-based optical reference light source: 7.18 × 10^8^ photons s^−1^ at 632 nm in the photon flux of the LED-based optical reference light source were detected at a signal of 8.60 × 10^7^ RLU on the CCD camera. Relative spectral response (inside figure) of the imaging system, normalized with respect to that at 632 nm. (**b**) Absolute bioluminescence imaging of the green-emitting (TK-Eluc) and red-emitting (SV40-SLR) luciferase-expressing cells. BLI was visualized by the BLI system, having been subjected to exposure for 10 min following the addition of 0.2 mM firefly luciferin, which was measured under the same conditions as that of the LED-based optical reference light source. The BLI of the selected cells was determined by the spectral photon flux (photons s^−1^ μm^−2^) as a total absolute optical signal.

**Step (1)** A schematic example of the calibration of the BLI system of the inverted microscope based on the LED-based optical reference light source signal is shown in Figure 4a. The photon flux of the LED-based optical reference light source (7.18 × 10^4^ photons s^−1^ at 632 nm) was used to calibrate the light scope system, which resulted in one RLU of this system being determined to be 0.13 photons at 632 nm. Second, the photon number of the optical signal was converted to 0.122 photons RLU^−1^ at 538 nm, which was the target color light output for adjusting the expressed luciferase from blue to red based on the relative spectral sensitivity of the camera. In this case, the light outputs were assumed to exhibit a linear relationship within the assumed range. 

**Step (2)** Optical images of the BLI of stable cell lines expressing TK-regulated green-emitting luciferase (TK-Luc) and SV40-regulated red-emitting luciferase (SV40-SLR) are shown in Figure 4b. For example, the optical signal of 513 RLU s^−1^ cell^−1^ in Cell#1 of TK-Luc was converted to 1.17 ± 0.14 × 10^4^ photons s^−1^ cell^−1^ as an absolute value. The optical signal of 944 RLU s^−1^ cell^−1^ in Cell#1 of SV40-Luc was converted to 8.00 ± 0.94 × 10^3^ photons s^−1^ cell^−1^ as an absolute value. 

Furthermore, the number of luciferase proteins was counted based on the quantum yield of the luciferin–luciferase reaction. Despite using different color luciferases, the light signal outputs could directly reflect promoter activities at the single-cell level. This implies that we can directly compare gene expression in different-colored luciferase-emitting cells or using different BLI systems.

## 6. Application of Bioluminescent Immunohistochemistry

Immunohistochemistry (IHC) is an important technique for sensitive detection systems, including colorogenic substrates, fluorescent probes, and BL probes [64,65,66,67,68]. IHC detects target molecules in cell or tissue sections using antigen-specific antibodies. However, IHC images are not yet suitable for quantitative analysis, despite their ability to quantify the signals of the immune assay. In an immune assay, the calibration curve between the amount of antigen and the optical signal of the antibody reaction can quantify the target molecule. For IHC, it is difficult to create a calibration histogram between the amount of antigen and the optical signal of the imaging. Among several techniques, BL-IHC has the potential to visualize target antigens quantitatively and rapidly [69]. In this case, it is important to construct a calibration curve between the amount of antigen and optical signal of the antibody-fused luciferase–luciferin reaction.

Protein chip technology was used to calibrate the amounts of antigen and optical imaging signals [70]. Protein microarray was used to spot the target antigen on a suitable slide using the same IHC procedure. In the microarray experiments, spotted slides were first incubated with blocking buffer and then with a diluted antibody-fused luciferase probe. After washing the slide twice or thrice with PBS and adding luciferin, the BLI system captured the light signal of the luciferin–luciferase reaction, creating a calibration curve between the antigen and antibody. The calibration curve revealed the amount of antigen in the BL-IHC images.

**Figure 5:**
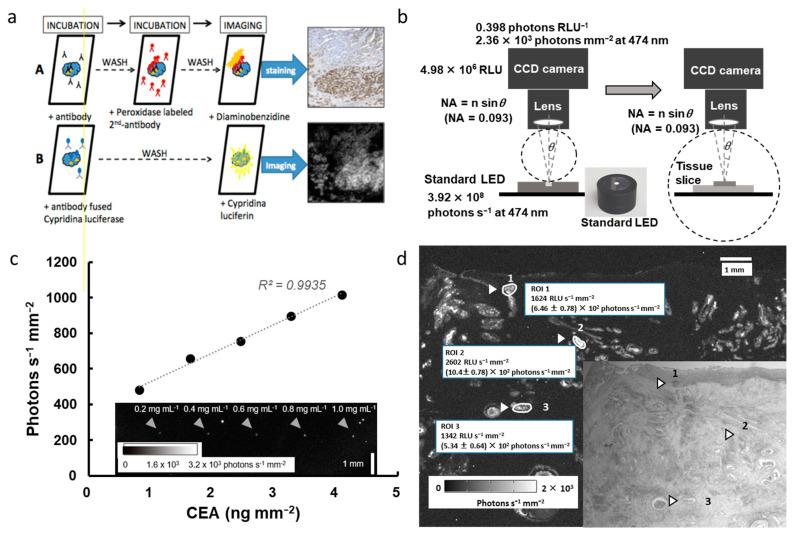
**Outline of bioluminescence immunohistochemistry from procedure to results (refs. [69,71]).** (**a**) Procedure for immunohistochemical staining of serial paraffin sections using indirect peroxidase labeling methods (A) and direct antibody-fused luciferase labeling (B). (**b**) Schematic illustrations of the setup and procedure for calibrating the bioluminescence imaging system based on the LED-based optical reference light source. At 474 nm, the photon flux of the LED-based optical reference light source contained 2.36 × 10^8^ photons s^−1^, corresponding to 4.98 × 10^6^ RLU on the CCD camera. (**c**) Calibration curve between antigen count and photon numbers in the antigen-dotted protein microarray. (**d**) Absolute bioluminescence imaging immunohistochemistry of surgical pathological specimens and light imaging (inside photo). Antigen distribution and determination of photon number of three randomly selected spots in the colon cancer (shown in white).

Figure 5a shows the procedure of IHC visualization, that is, immunohistochemical staining of serial paraffin sections using antibody-fused luciferase (B), in comparison with the peroxidase labeling procedure (A) [71]. The optical signal of BL-IHC can be absolutely counted by the imaging system, whereas the signal using the indirect secondary labeling system visualizes antigen distribution. This review also introduces an example of the calibration process of absolute BLI histochemistry, as follows:

**Step (1)** The calibration schematic process of the BLI system using the optical signal of the LED-based optical reference light source, which is characteristic of the total flux and light distribution on the stage of upright microscopy, is shown in Figure 5b. The photon flux of the LED-based optical reference light source (3.92 × 10^8^ photons s^−1^ at 474 nm) was used to calibrate the light scope system using CCD camera, resulting in one RLU of this system being determined to be 0.398 photons at 474 nm and converted to 2.36 × 10^8^ photons s^−1^ mm^−2^).

**Step (2)** An example of protein-chip imaging and the calibration curve between the target antigen and antibody-fused luciferase are shown in Figure 5c. The protein microarray containing the control antigen protein (Carcinoembryonic antigen, CEA protein) for 0.2–1.0 mg mL^−1^ per spot (~0.014 mm^2^) using a Protein Microarrayer Robot. Following incubation with blocking buffer, the spotted slides were incubated with antibody-fused luciferase. Finally, after washing, the antibody-fused luciferase and luciferin reaction visualized the optical signal of the protein microarray, which was approximately 480–1000 photons s^−1^ mm^−2^. The calibration curve between the antigen and photon numbers indicates a linear relationship ranging between 1.0 and 4.0 ng mm^−2^. 

**Step (3)** Optical images of BL-IHC of colon cancer cells using anti-CEA-fused luciferase are shown in Figure 5d. Based on the calibration curve, the RLU image was changed to an absolute light signal in the BLI image and was converted to the target antigen. For example, as an absolute value, the optical signal of 1,624 RLU s^−1^ mm^−2^ in ROI 1 of cancer tissue was converted to 6.24 ± 0.78 × 10^2^ photons s^−1^ mm^−2^. Based on the calibration curve shown in Figure 5c, the amount of CEA in ROI 1 was estimated to be approximately 1.8 μg. 

The optical signal of BL-IHC can be used to directly count the amount of the target antigen in the selected area using an optical reference light source. We can evaluate cancer stages absolutely if there is a relationship between the number of antigens and the cancer stage. This approach could pave the way for new avenues in quantitative diagnostic pathology.

## 7. Closing Remark: Open to Quantitative Biology by Quantifying the Optical Light Signal

Scientists have been content with the qualitative analysis of biological phenomes. However, they aim to determine the number of receptor proteins on the cell membrane or the number of ATP molecules in living cells. The light output can be converted into an absolute photon number based on an optical reference light source. The light output of the firefly luciferin–luciferase reaction is proportional to the amount of ATP in excess of luciferin and luciferase, allowing us to count the number of ATP molecules based on the QY of this reaction. Using antibody-fused luciferase, the light output depends on the amount of target antigen based on the calibration, resulting in counting of the number of target molecules. Furthermore, the absolute photon number can be used to count the number of molecules by using the QY of the luciferin–luciferase reaction. In conclusion, the absolute number of biological molecules can open up to the current quantitative biology and progress in understanding biological functions in vitro and in vivo.

## Figures and Tables

**Table 1:** Luminescence maxima and QYs for beetle luciferases.

Luciferase	λ_Max_ ^†^ (nm)	QY	±σ ^‡^
*Pyrearinus termitilluminans*, wild type	539	0.61	0.019
*Phrixothrix hirtus*, wild type	625	0.15	0.017
*Pyrocoelia miyako*, wild type	554	0.45	0.055
*Pyrocoelia miyako*, mutant N230S	606	0.21	0.0072
*Pyrocoelia miyako*, mutant S199T	559	0.48	0.056
*Pyrocoelia miyako*, mutant S200A	556	0.46	0.036
*Photinus pyralis*, native ^§^	566	0.48	0.039
*Photinus pyralis*, recombinant wild type	560	0.45	0.055

All values were measured at 24 °C and pH 8.0. The relative standard uncertainty of QY measurements was approximately 15%. † Maximum of the bioluminescence spectrum. ‡ Standard deviation of QY measurements. § Reported in ref. [33]. Others were in ref. [48].

## Data Availability

Not applicable.
